# *Staphylococcus aureus* Proteins Sbi and Efb Recruit Human Plasmin to Degrade Complement C3 and C3b

**DOI:** 10.1371/journal.pone.0047638

**Published:** 2012-10-11

**Authors:** Tina K. Koch, Michael Reuter, Diana Barthel, Sascha Böhm, Jean van den Elsen, Peter Kraiczy, Peter F. Zipfel, Christine Skerka

**Affiliations:** 1 Department of Infection Biology, Leibniz Institute for Natural Product Research and Infection, Biology, Jena, Germany; 2 University of Bath, Bath, United Kingdom; 3 Institute of Medical Microbiology and Infection Control, University Hospital Frankfurt, Germany; 4 Friedrich Schiller University Jena, Germany; Charité-University Medicine Berlin, Germany

## Abstract

Upon host infection, the human pathogenic microbe *Staphylococcus aureus* (*S. aureus*) immediately faces innate immune reactions such as the activated complement system. Here, a novel innate immune evasion strategy of *S. aureus* is described. The staphylococcal proteins surface immunoglobulin-binding protein (Sbi) and extracellular fibrinogen-binding protein (Efb) bind C3/C3b simultaneously with plasminogen. Bound plasminogen is converted by bacterial activator staphylokinase or by host-specific urokinase-type plasminogen activator to plasmin, which in turn leads to degradation of complement C3 and C3b. Efb and to a lesser extend Sbi enhance plasmin cleavage of C3/C3b, an effect which is explained by a conformational change in C3/C3b induced by Sbi and Efb. Furthermore, bound plasmin also degrades C3a, which exerts anaphylatoxic and antimicrobial activities. Thus, *S. aureus* Sbi and Efb comprise platforms to recruit plasmin(ogen) together with C3 and its activation product C3b for efficient degradation of these complement components in the local microbial environment and to protect *S. aureus* from host innate immune reactions.

## Introduction

*Staphylococcus aureus*, a persisting human pathogen, can cause a variety of diseases including skin infections, pneumonia, endocarditis, and sepsis [1], [2]. Upon infection, *S. aureus* is immediately recognized and targeted by innate immune responses, such as the complement system. Activation of complement by foreign surfaces (alternative pathway, AP), by antibodies (classical pathway, CP) or by mannan (lectin pathway, LP) causes microbial opsonization, leukocyte recruitment, and cell lysis. All three pathways lead to the cleavage of C3 and subsequent formation of anaphylatoxin C3a and opsonin C3b. C3a attracts and activates granulocytes; whereas C3b attaches covalently to the bacterial surface, amplifies complement activation, and thereby labels cells for phagocytosis. Furthermore, C3b deposition leads to inflammatory reactions and formation of the pore-forming terminal complement complex [3]–[5].

*S. aureus* evades the complement system by targeting C3 and the activity of C3 convertases [6]. At least three C3 binding proteins that exert complement inhibitory functions have been identified in *S. aureus*: staphylococcal immunoglobulin-binding protein (Sbi), extracellular fibrinogen-binding molecule (Efb), and the Efb homologue protein Ehp [7]–[9]. Sbi blocks the AP by induction of C3 consumption [10], [11] and Efb inhibits binding of factor B to C3b and blocks the C3 and C5 convertases [12], [13]. The interaction of Sbi or Efb with the thioester-containing domain (TED) in C3 induces a conformational change in the C3 molecule [11], [14]. In a tripartite complex with the human complement regulator factor H, C3 [14] or C3b [15] is degraded by factor I. Sbi binds C3 via its structural domains 3 and 4 [11], [15], which harbor a three-helix bundle motif and are structurally related to the C3 binding domain in Efb [14], [16].

Previous work demonstrated that *S. aureus* binds the human plasma protein plasminogen (PLG) to its surface and expresses a plasminogen activator, staphylokinase (SAK), that converts plasminogen to the serine protease plasmin (PL) [17], [18]. However, it was unclear how *S. aureus* binds plasminogen to target the host C3 molecule for complement evasion. Plasmin controls several processes such as fibrinolysis, wound healing and tissue remodeling. Thus pathogenic microbes often exploit the proteolytic activity of plasmin to degrade components of the extracellular matrix (ECM), as well as fibrinogen for dissemination in the host [19], [20]. Beside fibrinolytic activity, plasmin can cleave native C3 leading to the formation of the anaphylatoxin C3a [21]–[24] and C3b, which is subsequently degraded and inactivated to iC3b and C3c [25]–[28].

This report demonstrates that plasminogen, which is bound by the staphylococcal proteins Sbi and Efb, is converted to plasmin by *S. aureus* secreted SAK. Bound plasmin subsequently cleaves C3 and C3b that are simultaneously bound by Sbi and Efb. This proteolytic process are enhanced by the conformational changes Efb and presumably Sbi induce in C3 and C3b. In addition, plasmin was shown to degrade C3a that harbors chemoattractant and antimicrobial activities. Thus, recruitment and activation of plasminogen by *S. aureus* is highly coordinated to maximize complement inhibition at the level of C3.

**Figure 1:**
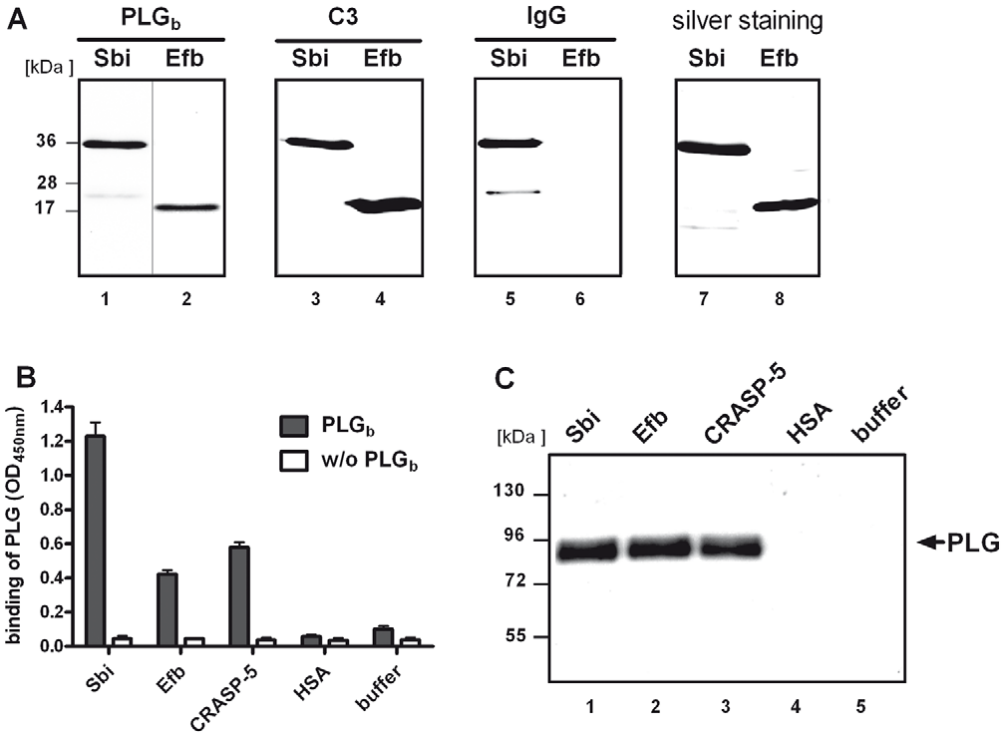
Plasminogen is recruited by Sbi and Efb. (A) Plasminogen bound to Sbi and Efb (lane 1 and 2). The accuracy of the recombinant staphylococcal proteins was confirmed as both, Sbi and Efb, bound to C3 (lane 3 and 4) and Sbi also bound IgG. Recombinant Sbi or Efb was separated by SDS-PAGE and either blotted to a membrane (lane 1–6) or silver stained (lane 7–8). Membranes were incubated with biotinylated PLG (PLG_b_), C3, or HRP- coupled IgG and bound proteins were detected as indicated. (B) Binding of plasminogen to Sbi or Efb was confirmed by ELISA. Bacterial proteins (equimolar) were immobilized and PLG_b_ was applied and detected using streptavidin-HRP. Plasminogen binding to Sbi and Efb was comparable to plasminogen binding to the borrelial protein CRASP-5. Plasminogen did not bind to HSA or the plate (buffer). Data represent mean values ± standard deviations from three independent experiments. (C) Plasminogen was recruited to Sbi and Efb. Non-labeled plasminogen was applied to immobilized bacterial proteins. Bound proteins were eluted, separated by SDS-PAGE and plasminogen (92 kDa) was detected by Western blot analysis. CRASP-5 bound plasminogen in contrast to HSA and buffer. A representative experiment out of three is shown.

## Materials and Methods

### Bacterial strains

*S. aureus* strain Newman was cultured on blood agar (Invitrogen) or in Luria broth at 37°C.

**Figure 2:**
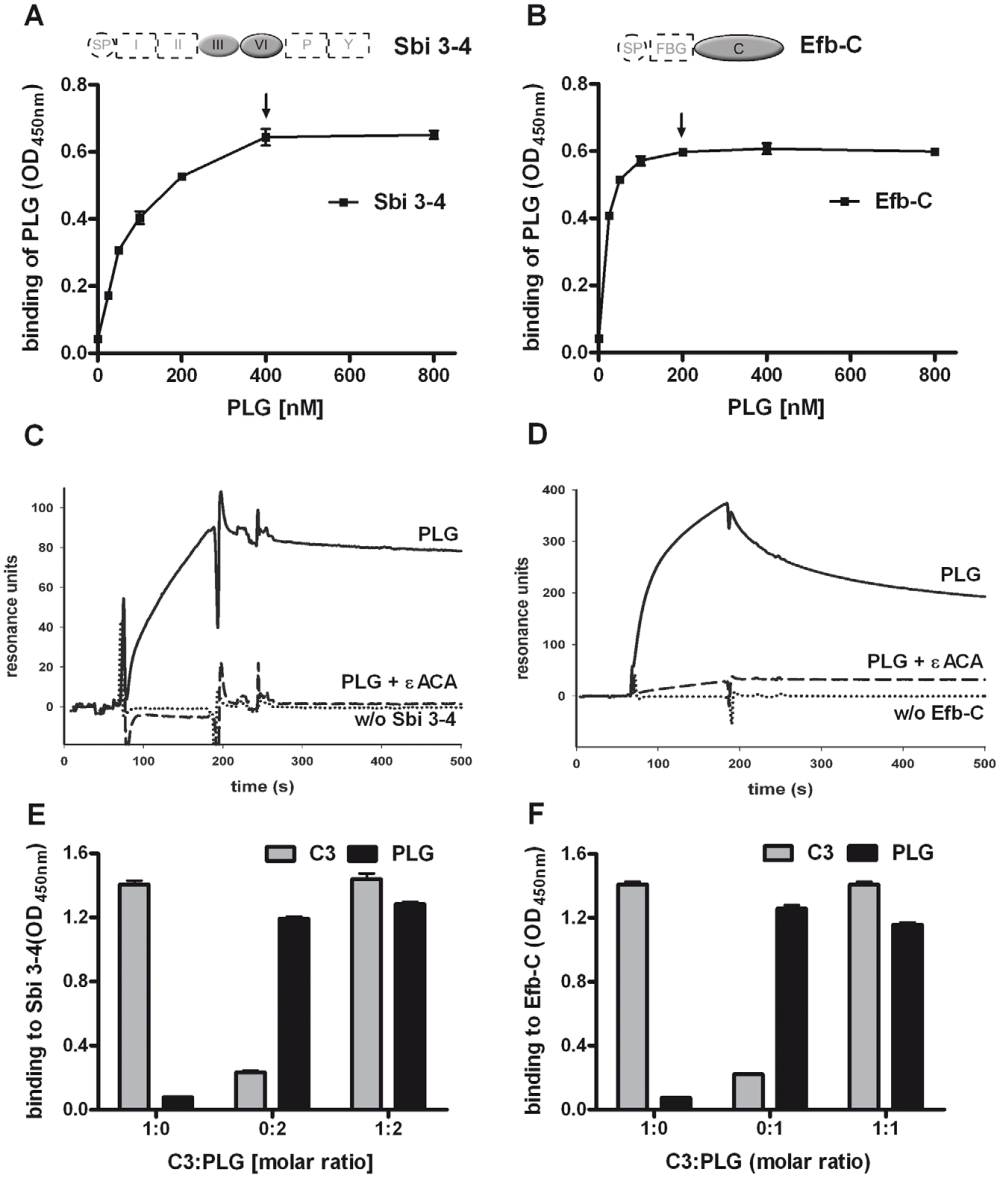
Characterization of the Plasminogen interactions with Sbi and Efb-C. (A+B) Plasminogen (PLG) bound to the fragments 3–4 of Sbi (A) and the C-terminus of Efb (B). Sbi 3–4 or Efb-C was immobilized and increasing amounts of plasminogen were added. Bound plasminogen was detected with specific antisera. Plasminogen concentrations showing saturated binding are marked by arrows. (C+D) Plasminogen (solid lines) associated with Sbi 3–4 (C) or Efb-C (D) was determined by surface plasmon resonance. Sbi 3–4 or Efb-C was immobilized and plasminogen was applied in fluid phase. Binding of plasminogen to Sbi 3–4 or Efb-C was inhibited by εACA (dashed line). (E+F) Plasminogen (black columns) and C3 (grey columns) bound simultaneously to Sbi 3–4 (E) and Efb-C (F). Sbi 3–4 or Efb-C was immobilized and C3 and plasminogen were added either alone or together (200 nM C3:200 nM PLG  = 1∶1 for EfbC, 200 nM C3:400 nM PLG = 1∶2 for Sbi3-4). Representative experiments out of three independent experiments are shown.

### Cloning, Expression and Purification of proteins

Recombinant Sbi 1–4 (subsequently referred to as Sbi) and Sbi 3–4 were expressed as previously described [15]. Efb and Efb-C were expressed using the *E.coli* pET200/D-TOPO expression system (Invitrogen) and Efb forw: CACCTCTTTATTAACATTAGCGGCAATAG and Efb rev: TTATTTAACTAATCCTTGTTTTAATACA, or Efb-C forw: CACCACTGATGCAACTATTAAAA and Efb-C rev: TTATTTAACTAATCCTTGTTTTAATACA primers for amplification. Sbi 1–2 and SAK were cloned into *E.coli* pET101/D-TOPO expression vector (Invitrogen) using Sbi 1–2 forw: CACCATGACAACTCAAAACAACTACGTAAC and Sbi 1–2 rev: ATTTTGACGTTCTTTAGCTTTAGAAGATTGTACTG, or SAK forw: **CACC** *ATG*CTCAAAAGAGGTTTATTATTTTTAAC and SAK rev: TTTCTTTTCTATAATAACCTTTGTAATTAAGTTG primers. Expression and purification was performed according the user's manual. Affinity chromatography with Ni-NTA-Agarose (GE Healthcare) was used as previously described [15]. CRASP-5 of *B. burgdorferi* was expressed and purified as previously described [29] [29] [29] [29]. Plasma purified plasminogen (Haemochrom Diagnostica) was biotinylated with Sulfo-NHS-LC-Biotin (Thermo Scientific) according to manufacturer's instructions. However a 10 fold molar excess of biotin was used. Unbound biotin was removed by Zeba^TM^ Desalt Spin Columns (Pierce).

**Figure 3:**
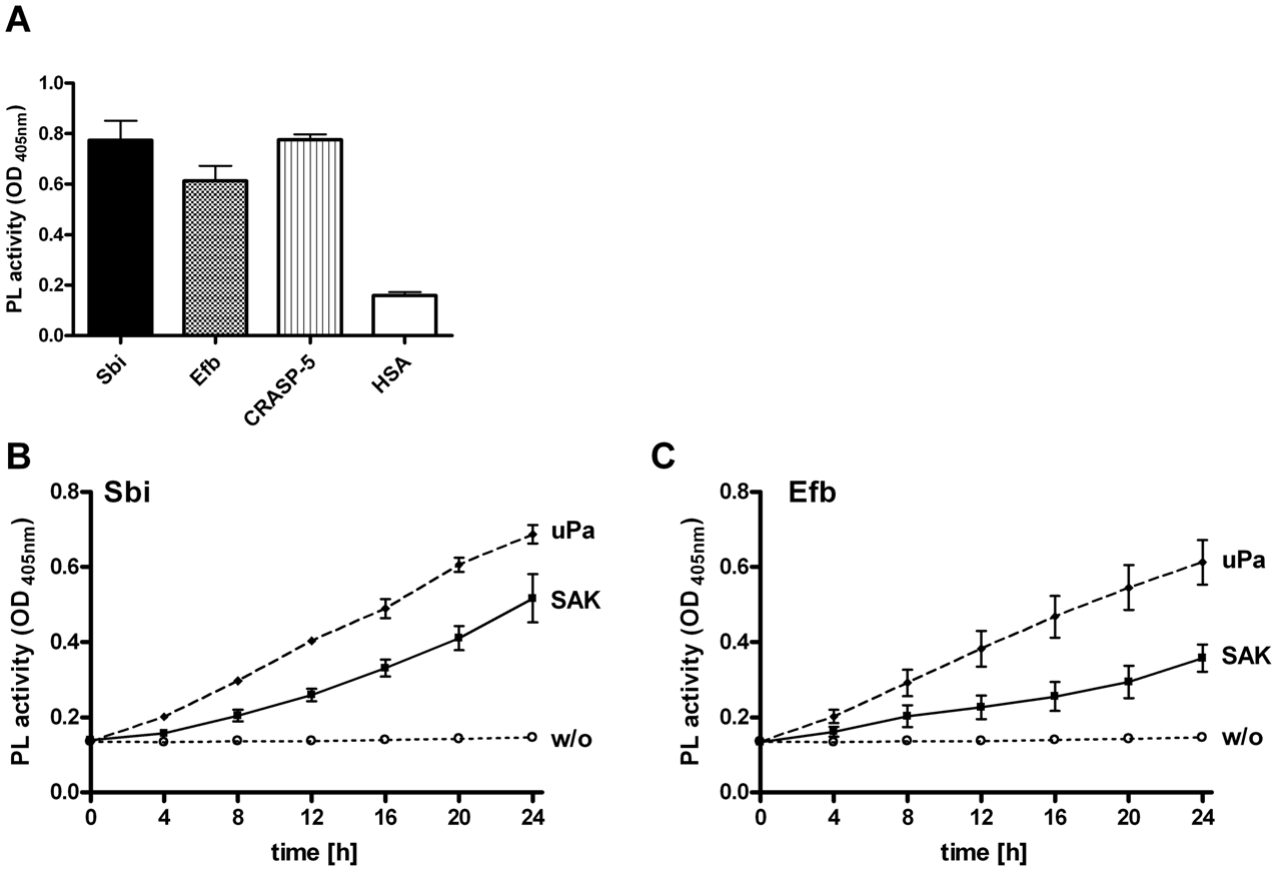
Plasminogen bound to Sbi or Efb is converted to plasmin. (A) Plasmin (PL) bound to Sbi, Efb, and to the borrelial CRASP-5 converted a chromogenic substrate as followed by measuring absorbance at 405 nm after 24 h. Equimolar amounts of bacterial proteins were immobilized and incubated with plasminogen (PLG). After washing, the activator uPA was applied together with the chromogenic substrate S-2251. Human serum albumin (HSA) had no effect. (B+C) Plasmin generation of plasminogen bound to Sbi (B) and Efb (C) by uPa or staphylokinase (SAK). The plasmin activators uPa and SAK or no activator (w/o) were applied to plasminogen bound to Sbi or Efb together with the chromogenic substrate. Conversion of the chromogenic substrate was determined at various time points. Data represent mean values ± standard deviations from three independent experiments.

### Ligand blot

Staphylococcal proteins were separated by SDS-PAGE and transferred onto a nitrocellulose membrane. After blocking with 1% BSA, 4% milk powder, 0.1% Tween20 in PBS for 1 h at RT, the membranes were incubated with 1 µg/ml biotin-coupled plasminogen (PLG_b_) or C3 (CompTech) overnight at 4°C. Bound PLG_b_ was detected with Streptavidin-Peroxidase (1∶1000; Sigma), C3 with anti-C3-Fab-HRP (1∶1000; Protos Immunoresearch), and to assess IgG-binding, rabbit anti-goat-HRP (1∶1000; DAKO) was applied. All blots were developed using the ECL-substrate (AppliChem).

**Figure 4:**
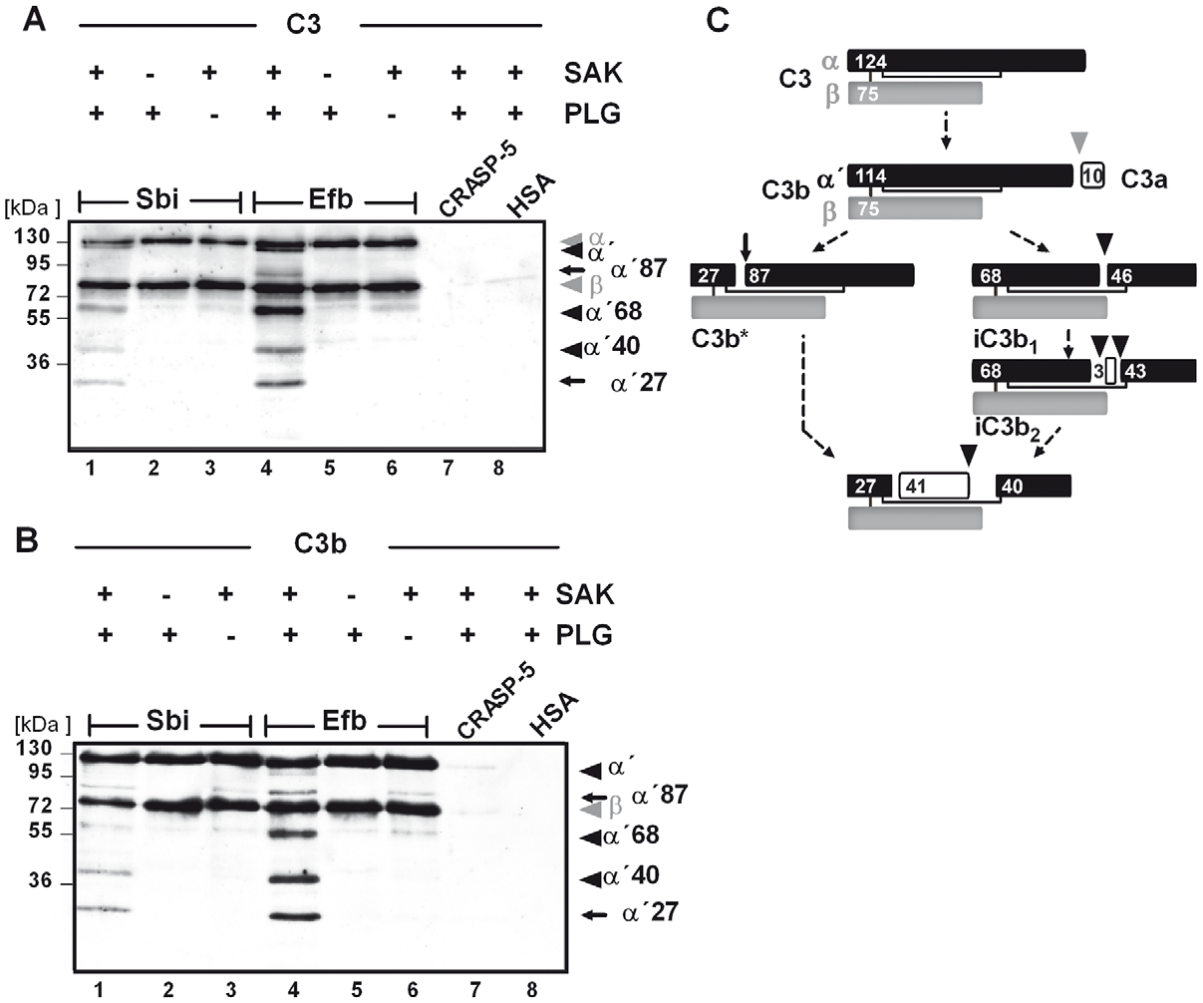
Plasmin complexed with C3(b) and Sbi or Efb degrades C3 and C3b within the complex. Plasmin degraded C3/C3b within the Sbi:plasmin:C3/C3b or Efb:plasmin:C3/C3b complexes (lane 1 and 4) and C3/C3b cleavage products appeared (marked with arrows). Bacterial proteins were immobilized (equimolar) and plasminogen was added together with C3 (A) or C3b (B). The activator SAK was applied and C3/C3b cleavage was followed by Western blot analysis using anti-C3-HRP (Fab). The mobility of the α (α ´) and β chains of C3/C3b and the cleavage products are indicated by arrows. CRASP-5 and HSA had no effect on cleavage (lane 7 and 8). Data shown are representative of three independent experiments. (C) Proposed schematic model of the C3 cleavage products generated by plasmin modified after [25], [32]. The C3 dg cleavage product is not recognized by the C3-antibodies used in this study.

### Enzyme linked Immunosorbent Assay (ELISA)

Bacterial proteins were immobilized (equimolar) onto a microtiter plate (Maxisorb, Nunc) blocked with 0.4% gelatin in DPBS for 2 h at 37°C, PLG_b_ was added for 1.5 h at 37°C, and bound proteins were detected with Streptavidin-Peroxidase (1∶1000) for 1 h at RT. The reaction was developed with TMB (KEM EM TEC), stopped by addition of 0.25 M H_2_SO_4_ and measured at 450 nm.

**Figure 5:**
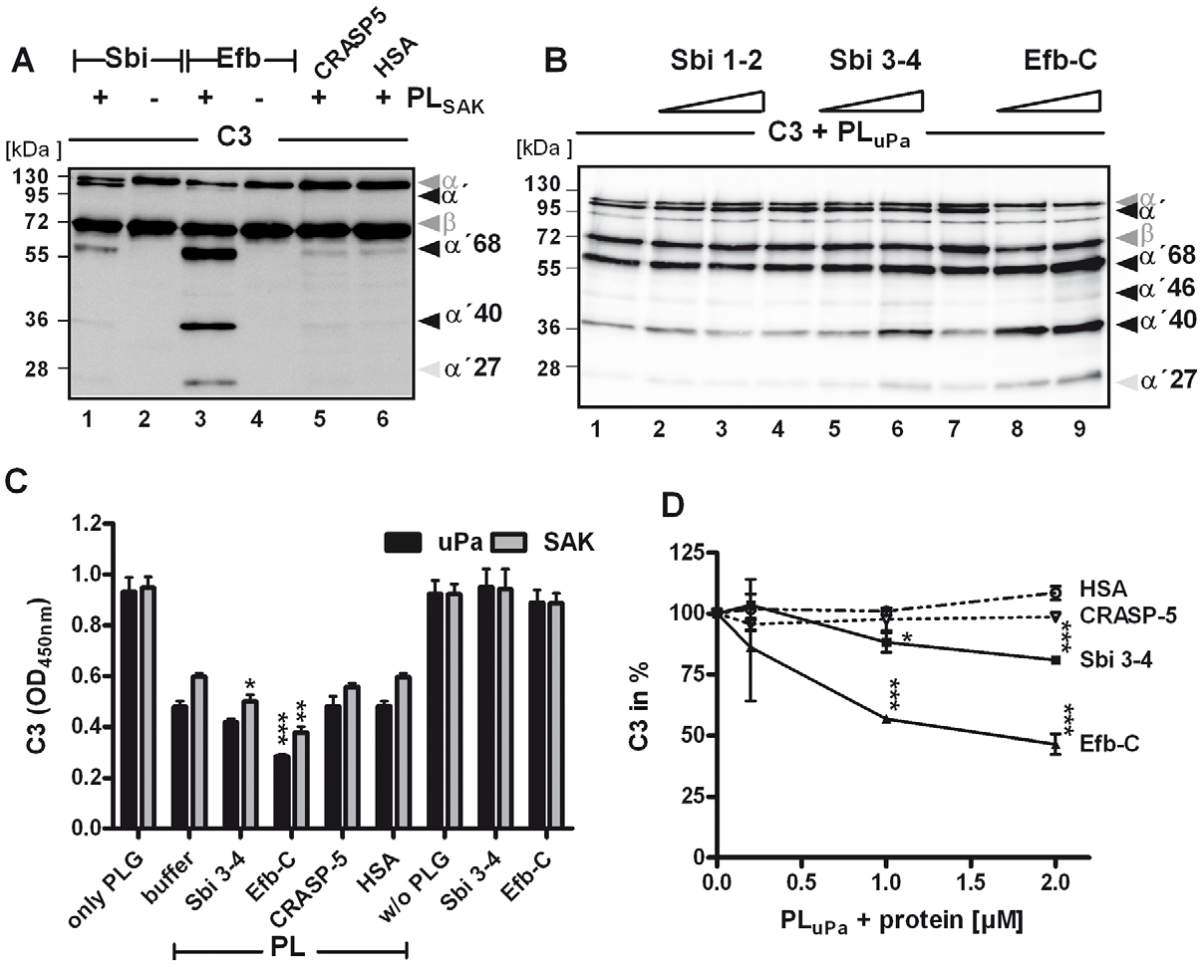
Plasmin mediated cleavage of C3 is enhanced by Sbi and Efb. (A) In the presence of Sbi and Efb (lane 1 and 2) the C3 α-chain decreased and C3 cleavage products (indicated by arrows) appeared. CRASP-5 did not show this effect. C3 was incubated with SAK-activated plasmin (PL_SAK_) in the presence of Sbi, Efb, CRASP-5, or HSA. Samples were separated by SDS-PAGE and C3 cleavage products were visualized using Western blot analysis. (B) A similar assay was performed with uPa as activator and this time the fragments Sbi 1–2, Sbi 3–4, and Efb-C were assayed. Sbi 3–4 (lane 5 and 6) and Efb-C (lane 8 and 9) enhanced the plasmin mediated C3 cleavage as seen by the appearance of small cleavage products. The IgG-binding Sbi 1–2 (mobility of 17 kDa) did not affect the C3 cleavage pattern (lane 2 and 3). Polyclonal C3 antiserum was used for detection. (C) In the presence of Sbi 3–4 and Efb-C the plasmin mediated C3 proteolysis was enhanced as measured by ELISA. C3 was immobilized and plasmin (activated by uPa or SAK) together with Sbi 3–4 or Efb-C were added. C3 deposition was detected after 3 h with C3a antiserum. CRASP-5 and HSA did not influence plasmin cleavage of C3. (D) Sbi 3–4 and Efb-C significantly enhanced C3 degradation by plasmin in a dose dependent manner. The C3 amount after incubation with plasmin was set to 100%. Data in C and D are mean values of three independent experiments; error bars indicate standard deviations. *p<0.05; **p<0.01 and ***p<0.001.

For dose-dependent binding of PLG to Sbi 3–4/Efb-C, 5 µg/ml Sbi 3–4 or Efb-C were immobilized to a microtiter plate, blocked with Blocking Solution I (AppliChem) for 2 h at 37°C and 25–800 nM PLG were added for 2 h at 37°C. Bound proteins were detected with anti-plasminogen (Acris Antibodies; 1∶1000) in Cross Down Buffer (AppliChem) and anti-goat HRP (1∶2000). In competition assays Sbi 3–4 or Efb-C were immobilized and incubated with concentrations of C3 (200 nM) and plasminogen (200 for Efb-C and 400 nM for Sbi 3–4) that saturate binding sites. C3 binding was detected using polyclonal anti-C3-antibody (CompTech; 1∶1000) and plasminogen binding with polyclonal plasminogen-antiserum.

**Figure 6:**
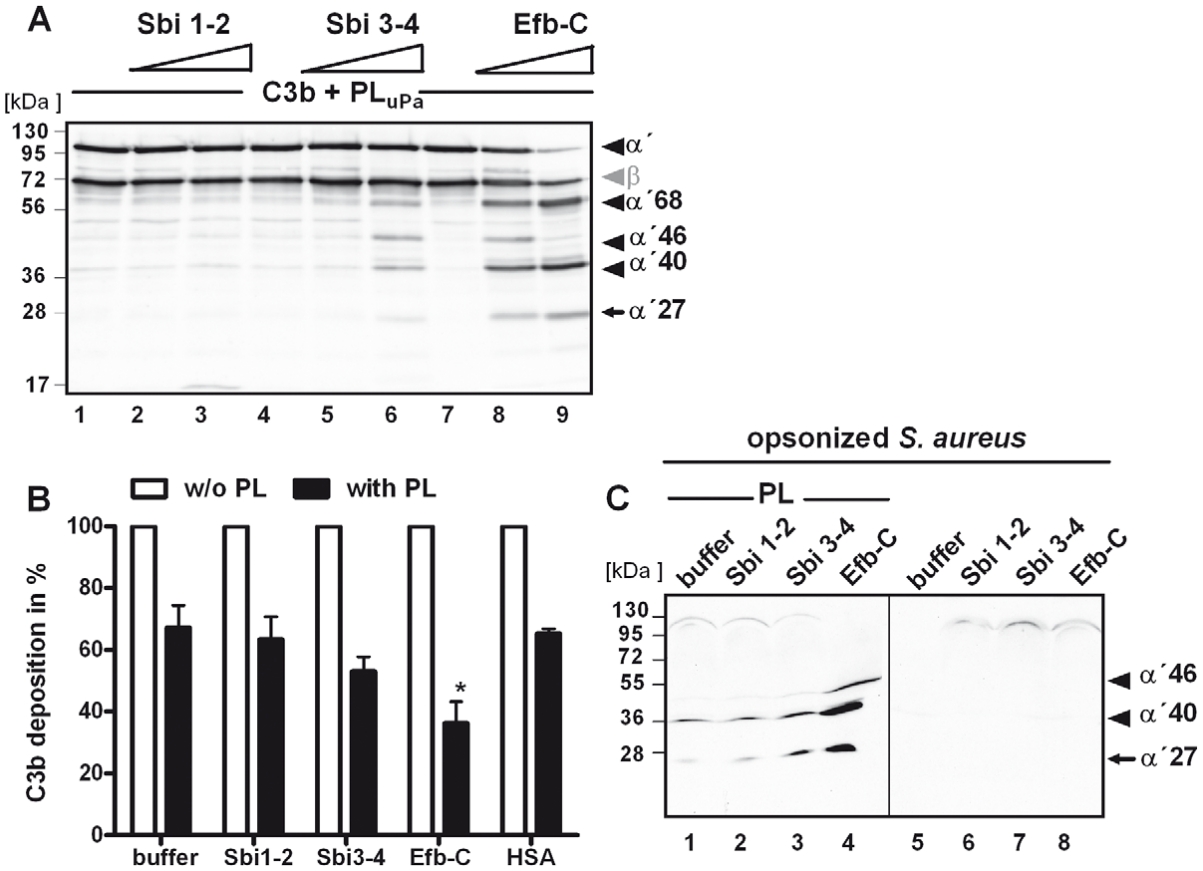
Plasmin cleavage of C3b is enhanced by Sbi and Efb. (A) Sbi 3–4 (lane 5–6) and Efb-C (lane 8–9) enhanced cleavage of C3b by plasmin, as seen by the accumulation of C3b cleavage products. Sbi 1–2 did not influence C3b degradation (lane 2–3). C3b was cleaved by plasmin in the presence of Sbi 1–2, Sbi 3–4, and Efb-C. (B+C) Sbi 3–4 and Efb-C enhanced the anti-opsonic activity of plasmin. C3b was deposited on *S. aureus* and Sbi 1–2, Sbi 3–4, Efb-C, or HSA was incubated in the absence or presence of plasmin. Remaining C3b fragments on *S. aureus* were measured by flow-cytometry. The C3b amount in the absence of plasmin was set to 100%. Data represent mean values ± standard deviations of five independent experiments. *p<0.05. (C) In parallel the supernatants were separated by SDS-PAGE and analyzed by Western blotting using anti-C3-HRP (Fab). Sbi 1–2 did not influence the amount of C3b-fragments after plasmin degradation (lane 1). Sbi 3–4 (lane 2) and Efb-C (lane 4) increased the plasmin degradation and more C3b cleavage products were detected.

### Combined ELISA-Western blot analysis (CEWA)

Combined ELISA-Western blot analysis was performed according to Haupt *et al* [15]. Briefly, equimolar amounts of Sbi, Efb, CRASP-5, and HSA were immobilized onto a microtiter plate. After blocking with gelatin, 50 nM plasma purified plasminogen was added and incubated overnight at 4°C. Bound plasminogen was eluted using SDS buffer, separated by SDS-PAGE, and analyzed by Western blotting using plasminogen antiserum (1∶2000). *Borrelia burgdorferi* CRASP-5 protein was included as a positive control and HSA (Cleveland, Ohio) as a negative control.

**Figure 7:**
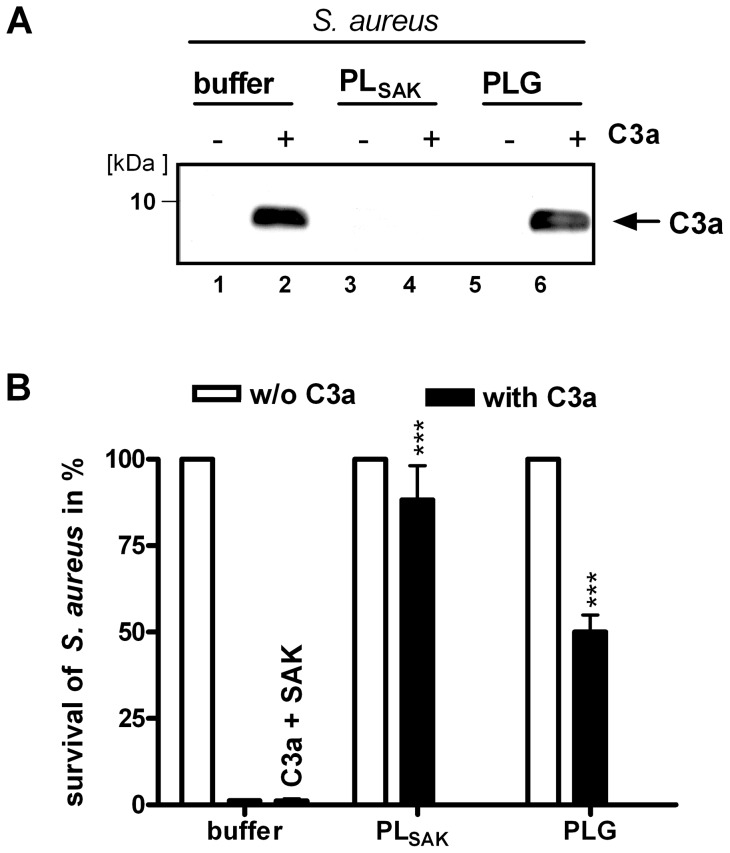
Plasmin degrades C3a. (A) C3a was completely degraded by plasmin; plasminogen had a weak degrading effect. *S. aureus* was incubated with C3a in the presence of plasmin (PLG+SAK) (lane 4) or plasminogen alone (lane 6). The supernatants were analyzed by SDS-PAGE and Western blot analysis. (B) Plasmin increased the survival of *S. aureus* to 85% and plasminogen alone to 50%. In parallel *S. aureus* treated with C3a and or plasmin(ogen) was cultivated overnight on LB agar plates. CFUs were counted and the survival without C3a was set to 100% (white columns). Data represent mean values ± standard deviations from three independent experiments. ***p<0.001.

### Surface plasmon resonance (SPR)

Binding of plasminogen to Sbi 3–4 or Efb-C was analyzed by surface plasmon resonance (Biacore 3000, AB) at 25°C in 150 mM PBS. Sbi 3–4 or Efb-C was immobilized on a CMD 500 M sensor chip (Xantec) by standard amino coupling chemistry following the manufacturer's protocol. Plasminogen (400 nM) was injected at a constant flow rate of 5 µl/min.

### Plasmin activity assay

Plasmin activation was determined by hydrolysis of the PL-specific substrate S-2251(D-valyl-leucyl-lysine-ρ-nitroanilide dihydrochloride; Haemochrom Diagnostica). Therefore Sbi, Efb, CRASP-5, or HSA were immobilized (equimolar) onto a microtiter plate. After blocking with 0.4% gelatin, 0.2 µM plasminogen was incubated at 4°C overnight. Following washing with PBS +0.05% Tween20, uPa (Haemochrom Diagnostica; 0.08 µg/ml) or recombinant SAK (1 µg/ml) were added together with the chromogenic substrate S-2251 dissolved in reaction buffer (64 mM Tris, 350 mM NaCl, 0.01% Triton-X; pH 7.5). PL activity was recorded at 4 h intervals at 405 nm (SpektraMax 190; Molecular Devices).

### Antimicrobial assay

C3a (1 µM) (CompTech) was incubated with *S. aureus* (3×10^4^) in 10 mM Tris-HCL with 5 mM Glucose (pH 7.4) with or without 5 µg PLG and/or 1 µg SAK at 37°C for 2 h with agitation. Bacteria were diluted 1∶5, 20 µl were plated onto a LB plate in triplicate, and incubated overnight at 37°C. Additionally the supernatant was separated by SDS-PAGE and analyzed by Western blotting with polyclonal anti-C3a antibody (1∶1000; CompTech).

### C3/C3b degradation by Sbi/Efb bound PL

Plasminogen (0.2 µM) together with either 0.04 µM C3 or 0.04 µM C3b were incubated with immobilized Sbi, Efb, CRASP-5, or HSA overnight at 4°C. After extensive washing 0.5 µM SAK or 0.06 µM uPa were added for 3 h at 37°C. Samples were reduced with Roti-Load for 5 min at 95°C, separated by SDS-PAGE and transferred onto a nitrocellulose membrane. C3/C3b and their cleavage products were detected with HRP-conjugated anti-C3-Fab antiserum (1∶1000).

### C3/C3b degradation by plasmin in fluid phase

0.04 µM C3 or C3b with either 0.15 µM plasminogen plus 0.04 µM uPa or 0.2 µM PLG plus 0.5 µM SAK were incubated in the absence or presence of Sbi, Efb, CRASP-5, or HSA (each 1 µM) for 3 h at 37°C. Additionally the same assays were performed using 0.2 or 2 µM of the fragments Sbi 1–2, Sbi 3–4, and Efb-C. Samples were analyzed as described above.

### C3 ELISA

C3 (0.04 µM) was immobilized to a microtiter plate at room temperature, after blocking with 0.4 % gelatin, 0.15 µM plasminogen with 0.04 µM uPa or 0.2 µM plasminogen with 0.5 µM SAK incubated for 3 h at 37°C. Sbi 3–4, Efb-C, CRASP-5, or HSA (each1 µM) were added in the presence or absence of plasmin. After extensive washing bound C3 was detected using C3a antiserum supplemented with 15 µg/ml aprotinin (to inhibit plasmin) and HRP-coupled antiserum at 4°C.

### C3b deposition assay

*S. aureus* was heat inactivated for 15 min at 72°C and incubated with 5% serum in HEPES buffer (20 mM Hepes, 140 mM NaCl_2_, 5 mM CaCl_2_, 25 mM MgCl_2_; pH 7.4) for 20 min at 37°C. Then bacteria were incubated with Sbi 1–2, Sbi 3–4, Efb-C, or HSA (each 2 µM) in the absence or presence of 0.15 µM plasmin (Calbiochem) for 2 h at 37°C. Supernatants were reduced with Roti-Load, separated by SDS-PAGE and analyzed by Western blotting using anti-C3-HRP. Bacteria surface-bound C3b was measured by flow cytometry with FITC-labeled anti-C3-Fab (Protos Immunoresearch).

### Statistical analysis

Significant differences between two groups were analyzed by the unpaired Student's t-test. Values of *p<0.05, **p<0.01, ***p<0.001 were considered as statistically significant.

## Results

### Plasminogen binds to Sbi and Efb

Sbi and Efb are C3 and C3b binding proteins. In order to investigate whether Sbi and Efb also recruit plasminogen, binding of plasminogen to recombinant Sbi and Efb was tested. The accuracy of recombinant Sbi and Efb was confirmed as both molecules showed binding to C3 and in addition Sbi bound to IgG (Fig. 1). Immobilized Sbi or Efb was incubated with biotin-labeled plasminogen (PLG_b_) and binding was assayed by ELISA. Plasminogen bound to both staphylococcal proteins Sbi and Efb. Plasminogen binding to Sbi and Efb was similar to the previously identified plasminogen ligand CRASP-5 of *Borrelia burgdorferi* [30], [31], (Fig. 1A+B). To exclude non-specific binding of plasminogen via its biotin-label, binding of unlabeled plasminogen to Sbi or Efb was tested using a combined ELISA-Western blot assay. Plasminogen was added to immobilized Sbi or Efb, washed, and all surface bound proteins were separated by SDS PAGE and immunoblotted for the detection of plasminogen. Also free plasminogen bound to Sbi, Efb, and CRASP-5 (Fig. 1C).

### Characterization of the PLG:Sbi and PLG:Efb interactions

To determine the Sbi and Efb domains responsible for interaction with plasminogen, plasminogen binding to the structurally related domains Sbi 3–4 and Efb-C in Sbi and Efb, respectively, was assayed. Plasminogen added to the immobilized domain fragments bound to both Sbi 3–4 (Fig. 2A) and Efb-C (Fig. 2B) in a dose-dependent manner. These binding interactions were also followed in real time by using surface plasmon resonance. Plasminogen added in fluid phase bound to immobilized Sbi 3–4 or Efb-C, confirming the interaction between plasminogen and these specific Sbi and Efb domains. To investigate whether plasminogen binding to Sbi and Efb occurs via lysine residues, εACA, a lysine analog which blocks lysine residues, was used in binding assays. εACA interfered with plasminogen binding to both Sbi 3–4 and Efb-C, demonstrating that lysine residues are involved in the interactions of these bacterial proteins with plasminogen.

To determine whether plasminogen and C3 bind simultaneously to Sbi and Efb, each plasma protein was added to immobilized Sbi 3–4 or Efb-C using concentrations of plasminogen (400 nM for Sbi 3–4 and 200 nM for EfbC) which saturate all binding sites. Plasminogen and C3 binding were measured in parallel. C3 binding to Sbi 3–4 and Efb-C, as well as plasminogen binding to Sbi 3–4 remained unchanged. Plasminogen binding to Efb-C was slightly reduced in the presence of C3 (Fig. 2E and 2F). The data indicate that plasminogen and C3 bind simultaneously to both staphylococcal molecules.

### Plasminogen bound to Sbi or Efb is activated to plasmin

To exert proteolytic activity, plasminogen needs conversion to plasmin by an activator. To proof whether plasminogen bound to Sbi or Efb is converted to plasmin. Plasminogen was bound to immobilized Sbi or Efb and treated with the human activator uPa. Plasmin generation was followed by cleavage of a plasmin-specific chromogenic substrate. Plasminogen-bound Sbi or Efb was activated by uPa to plasmin, as seen by the increased conversion of the substrate S-2251. Similarly, plasminogen bound to CRASP-5 was activated to the protease plasmin (Fig. 3A).

SAK is known to activate plasminogen via a non-proteolytic mechanism. To determine whether SAK is also able to activate Sbi- or Efb-bound plasminogen SAK was used in similar experiments instead of uPa. Also SAK activated plasminogen bound to Sbi or Efb, and the resulting plasmin cleaved the chromogenic substrate (Fig. 3B and C). In summary, plasminogen bound to Sbi and Efb is activated by both staphylococcal and human activators to form active plasmin.

### Plasmin bound to Sbi or Efb degrades C3 and C3b

After plasmin(ogen) was shown to form complexes with C3 and Sbi or Efb, the plasmin activity was assayed to cleave complexed C3. Plasminogen was bound together with C3 to immobilized Sbi or Efb and activated by the addition of SAK. C3 degradation was investigated by separating the reaction mixture by SDS-PAGE and Western blot analysis using anti-C3 Fab'-fragments. Multiple C3-degradation products with mobilities of 115, 87, 68, 40, and 27 kDa were generated indicating that Sbi- or Efb-bound plasminogen was activated by SAK to plasmin which subsequently cleaved bound C3 (Fig. 4A). In a similar assay performed with C3b, cleavage products with mobilities of 87, 68, 40, and 27 kDa appeared and demonstrated that complexed plasmin also degraded C3b (Fig. 4B). When CRASP-5 or HSA was used in these assays, instead of Sbi or Efb, no cleavage of C3 was observed which is explained by the fact that CRASP-5 and HSA do not acquire plasmin together with C3/C3b. Thus, plasmin complexed together with C3/C3b and Sbi or Efb degrades the complement proteins C3 and C3b.

### C3 degradation by plasmin is enhanced by Sbi and Efb

Upon binding, Efb changes the structural conformation of C3, leading to an increased susceptibility of C3 to degradation by trypsin [14]. To analyze whether C3 degradation by plasmin is also modulated by Sbi or Efb, C3 proteolysis by plasmin was compared in the presence or absence of Sbi or Efb. C3 degradation by plasmin was enhanced by both staphylococcal proteins, as demonstrated by the appearance of additional C3 cleavage products in Western blot analysis using anti-C3 Fab'-fragments for protein detection (Fig. 5A). In the presence of Sbi, C3 cleavage products with mobilities of 114 kDa (α'), 68 kDa, and weakly 40 kDa appeared and with Efb, degradation products with mobilities of 68, 40, and 27 kDa were detected. By contrast, proteolytic cleavage of C3 by plasmin was not affected after addition of CRASP-5 or HSA. To localize the Sbi and Efb domains responsible for the degradation-enhancing activities, fragments Sbi1-2, Sbi 3–4, and Efb-C were investigated in the same assay. C3 cleavage by plasmin was enhanced in the presence of Sbi 3–4 and Efb-C, but no enhancement was observed in the presence of Sbi 1–2 (Fig. 5B). To confirm these results, C3 proteolysis was also examined using ELISA. C3 was immobilized on a microtiter plate and plasmin (generated by uPa or SAK) was added together with the bacterial proteins or with HSA. After incubation, the plate was washed and the amount of bound C3 was determined. Smaller amounts of C3 were detected in reactions containing plasmin than that of those containing plasminogen. The plasmin-dependent reduction of C3 deposition was enhanced by the addition of Sbi 3–4 and especially Efb-C to the reaction mixture, but not by the addition of CRASP-5 or HSA (Fig. 5C). This enhancement by Sbi 3–4 or Efb-C was more clearly demonstrated by using an assay in which increasing amounts of the bacterial proteins were used (Fig. 5D). The results indicate that plasmin cleaves C3, and that the staphylococcal proteins Sbi or Efb enhance the cleavage by plasmin. The two domains responsible for the degradation-enhancing effect were fragments Sbi 3–4 and Efb-C, each of which contains a three-helix bundle motif.

### C3b degradation by plasmin is also enhanced by Sbi and Efb

Having shown that plasmin cleaves C3 more efficiently in the presence of Sbi or Efb, the same effect was analyzed for C3b. Plasmin-mediated C3b degradation was assayed using the deletion fragments Sbi 1–2, Sbi 3–4, or Efb-C, in the same assay as described for C3. The C3b degradation by plasmin was enhanced in the presence of Sbi 3–4 or Efb-C, as shown by the increase in C3b degradation products (Fig. 6A). However, the addition of Sbi 1–2 showed no enhanced degradative effect. To investigate whether Sbi and Efb also enhance plasmin activity on the bacterial surface, *S. aureus* was incubated with human serum to allow complement-mediated C3b deposition. The C3b-coated bacteria were then washed, incubated with plasmin plus Sbi 3–4 or Efb-C, and C3b deposition was analyzed by flow cytometry. Plasmin substantially reduced the C3b opsonization of *S. aureus* by about 37%. In the presence of Sbi 3–4, C3b deposition was further decreased to 50%; and in the presence of Efb-C, to 38% (Fig. 6B). In parallel analyses, supernatants containing the C3b degradation products were assessed by SDS-PAGE and Western blotting. C3b cleavage products with mobilities of 41 and 27 kDa were identified in the supernatants of those samples containing C3b-opsonized *S. aureus* treated with plasmin (Fig. 6C). Again C3b degradation was enhanced when plasmin cleaved surface-bound C3b in the presence of Sbi 3–4 and especially Efb-C (indicated by arrows), and the presence of Sbi 1–2 showed no effect. These results demonstrate that C3b degradation by plasmin is accelerated by interaction with the staphylococcal proteins Sbi and Efb on the bacterial surface.

### Plasmin degrades C3a

Plasmin cleaves C3 and generates C3a [23], [24] and degrades C3b and thereby inactivates the C3b molecule for complement C3b amplification [25], [28], [32]. As C3a exhibits antimicrobial activity [33], [34] and thus can kill *S. aureus*, we investigated whether *S. aureus* recruited plasmin also degrades C3a. Therefore, *S. aureus* was treated with C3a alone or with C3a together with plasmin activated by SAK. The supernatants were analyzed by SDS-PAGE and Western blotting using C3a antisera. Plasmin completely degraded C3a, as demonstrated by the absence of detectable levels of C3a (Fig. 7A). When plasminogen was added without SAK to *S. aureus*, the amount of C3a was decreased, which is explained by synthesis and secretion of SAK by *S. aureus* during incubation time and subsequent activation of plasminogen to plasmin. In parallel, C3a antimicrobial activity against *S. aureus* was analyzed in survival assays. C3a added to growing *S. aureus* killed the bacteria, but in the presence of C3a and plasmin (PLG+SAK) 85% of *S. aureus* survived. Plasminogen added without SAK resulted in 50% survival of the bacteria, and addition of SAK without plasminogen had no effect on the bacteria (Fig. 7B). Thus, we conclude that plasmin inhibits the bactericidal activity of C3a by degradation of the C3a molecule.

## Discussion

Presented here is a novel complement evasion strategy employed by *S. aureus*. Human plasminogen and C3/C3b are simultaneously bound by the microbial proteins Sbi and Efb. Recruited plasminogen remains accessible for the human activator uPa or the bacterial activator SAK for conversion to active plasmin. Plasmin bound to Sbi and Efb degrades and inactivates C3 and C3b. This degradation is enhanced by conformational changes exerted by Efb and to a lesser extent by Sbi on both, bound-C3 and bound-C3b. Moreover, we show that plasmin degrades C3a, and thus inactivates the antimicrobial activity of C3a. Consequently, Sbi and Efb-recruited plasmin inhibits complement cascade progression, opsonization, antimicrobial activities, and inflammatory reactions.

The importance of the complement system in defense against *S. aureus* infections is reflected by the multiple strategies this human pathogen has developed to circumvent the host complement attack. Thereby, *S. aureus* preferentially targets the central complement component C3 to inhibit the auto-amplification loop of the alternative pathway [7], [35]. One of these strategies is the expression of small proteins that bind the C3 convertase, like SCIN [36], [37], or C3/C3b such as Efb [38], [39] and Sbi [11], [15]. Additionally, *S. aureus* recruits complement regulators from human plasma like factor H that acts as cofactor for the serine protease factor I to cleave C3b [15]. Here, we show that plasminogen is recruited by Sbi and Efb. To date, three staphylococcal plasminogen binding proteins have been described: inosine 5′-monophosphate, α-enolase, and ribonucleotide reductase [17]. However, in contrast to these plasminogen ligands, Sbi and Efb bind C3/C3b in addition to plasminogen. Plasminogen-binding is mediated by the domains Sbi 3–4 in Sbi and Efb-C in Efb. Although the sequence identity between Sbi 3–4 and Efb-C is rather low (only 19%), both fragments contain a three-helix bundle motif [16], in which amino acids of the α2-helixes contribute to C3d binding [10], [12]. Plasminogen and C3 bind simultaneously to Sbi 3–4 or Efb-C and are located closely to each other, which results in accelerated degradation of C3/C3b, once plasminogen is converted to plasmin. The C3/C3b degradation-enhancing activity of Efb-C is more pronounced as compared to Sbi 3–4. This difference is likely based on the higher binding affinity of C3d to Efb than Sbi (K_D_ [Efb-C:C3d]  = 0.8 nM, K_D_ [Sbi 3–4:C3d]  = 1.4 µM) [11], [14].

Plasmin cleaves C3b at several sites and generates C3c and C3d [25]–[28]. The interactions of plasminogen with both staphylococcal proteins Sbi or Efb depend on lysine residues. This is the same method of plasminogen binding observed in other microbial proteins such as PE from *H. influenzae* [27], CRASP-1 to -5 from *B. burgdoferi* [30], [31], Pra1 [40] and Gpm1 from *C. albicans* [41], and indicates a similar binding strategy, which ensures activation of plasminogen to plasmin for tissue evasion and complement escape.

Complexed with C3 and Sbi or Efb, plasminogen remains accessible for the staphylococcal activator SAK and the human activator uPa to be converted to plasmin. More than 67% of *S. aureus* strains express the *sak* gene and produce the non-proteolytic staphylokinase [42]. In addition, the pathogen can enhance uPa production in mammalian cells [43] or activate conversion of pro-uPa to uPa by metalloprotease aureolysin [44] to generate plasmin. Plasmin-mediated C3b degradation is accelerated by Sbi or Efb. These functions are mediated by the C3 binding domains Sbi 3–4 and Efb-C, and concur with studies showing that Efb-C-binding to C3 and C3b leads to a conformational change in C3 which enhances proteolytic cleavage of C3 by trypsin and factor I [14]. Thus, a similar effect of Efb-C on C3 for plasmin degradation is anticipated.

Plasmin has also been described as a complement activator, because it cleaves C3 to C3a and C3b [21]–[24]. However, as shown here and in previous studies, plasmin further degrades C3b and demonstrates anti-opsonic as well as complement–inhibitory activity [25]–[28]. Here, we show for the first time that purified C3a is also degraded by plasmin, and that in such cases, antimicrobial activities against *S. aureus* are decreased. These results are in accordance with those of Amara *et al*, who observed that while plasmin generates C3a, the C3a amount decreased when plasmin concentrations increased [24]. Thus, binding of plasmin by *S. aureus* helps the pathogen inactivate host antimicrobial activities and obviously represents a further survival strategy.

The powerful inhibitory effect of acquired plasmin(ogen) against several immune defense mechanisms apparently explains why many pathogenic microbes attach human plasminogen to their surface and activate plasminogen to plasmin. *S. aureus* is remarkably effective, using several plasmin activation pathways and expressing at least five plasminogen binding proteins. Among these proteins Sbi and Efb act like an amplifier of plasmin-mediated C3 cleavage to reduce local complement activity.
